# Secondary Malignant Transformation of Giant Cell Tumor of Bone: Is It a Fate?

**DOI:** 10.30699/IJP.14.2.165

**Published:** 2019-06-10

**Authors:** Sajjadeh Movahedinia, Tina Shooshtarizadeh, Hassan Mostafavi

**Affiliations:** 1 *MD-MPH, AP/CP, Pathology and Stem Cell Research Center, Afzalipour School of Medicine, Kerman University of Medical Sciences, Kerman, Iran*; 2 * Department of Pathology, School of Medicine, Iran University of Medical Sciences, Tehran, Iran *; 3 *MD, AP/CP, Department of Pathology, Shafa Yahyaean Orthopedics Hospital, Iran University of Medical Sciences, Tehran, Iran*; 4 *MD, Department of Radiology, Iran University of Medical Sciences, Tehran, Iran*

**Keywords:** Giant cell tumor, Bone, Malignant Tumor, Cell Transformation, p53 Genes

## Abstract

The malignant transformation of conventional giant cell tumor of bone (GCTOB) is rare and usually occurs with irradiation. Here we report two neglected cases of conventional GCTOB with spontaneous malignant transformation at 11 and 16 years after initial diagnosis. In the former case, the patient refused to receive any treatment following the incisional biopsy, and in the latter, the first recurrence that occurred 5 years after initial treatment, was neglected. Although rare, the occurrence of sarcomatous changes in these cases indicates that secondary malignant transformation may be part of the natural course of this tumor. In addition, in both cases, immunohistochemistry showed diffuse and strong p53 expression in the malignant tumor but not in the primary lesion. It suggests that p53 overexpression may play a key role in the malignant transformation of GCTOB and that investigating for p53 expression in recurred lesions may help in predicting cases of giant cell tumor, prone to malignant transformation.

## Introduction

Giant cell tumor of bone (GCTOB) is a benign locally aggressive neoplasm which accounts for 5% of resected primary and 20% of benign bone tumors (1). Primary malignant GCTOB, defined as a de novo high-grade sarcoma that arises concurrently with a benign GCT in the same lesion, is extremely rare (2). The secondary malignant transformation of a conventional GCT is more common and has long been supposed to be an occasional complication following radiation therapy, but rare cases with spontaneous sarcomatous transformations have been reported (3-6). Mechanisms of the malignant transformation of GCT remain unclear. A few studies suggest the possible role of *p53* mutation in local recurrence and sarcomatous change of GCTOB [7-9]. This report presents two rare cases of untreated GCTOB with spontaneous malignant transformation at 11 and 16 years following initial curettage. We performed the immunohistochemical study for p53 overexpression in these cases to identify the potential role of p53 alterations in the malignant transformation of GCTOB.

## Case Report

**Case 1**

**Figure 1:**
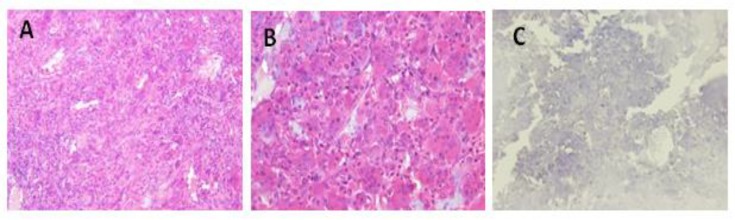
a 22-year-old male with right scapula giant cell tumor of bone (GCTOB), microscopic slides show the classic osteoclast-like giant cells and mononuclear cells (hematoxylin-eosin (H & E) stain, ×200) (A). High power view shows numerous multinucleated giant cells among stromal cells with similar nuclear characteristics without distinct cytoplasmic borders (H & E stain, ×400) (B). Staining with p53 antibody revealed no reaction neither in mononuclear tumor cells nor in multinucleated giant cells (immunohistochemistry, ×400) (C)

 A 33-year-old man was admitted to our center in December 2012 with progressive pain and swelling of right scapula leading to impaired function of the right shoulder. He reported a primary lesion in the same site, occurred 11 years ago in August 2001, which had been diagnosed as a conventional GCTOB on incisional biopsy. Microscopic evaluation of the first surgical specimen had shown numerous multinucleated giant cells in a background of mononuclear cells with plump vesicular nuclei, similar to those of multinucleated ones (Figure 1).

**Figure 2:**
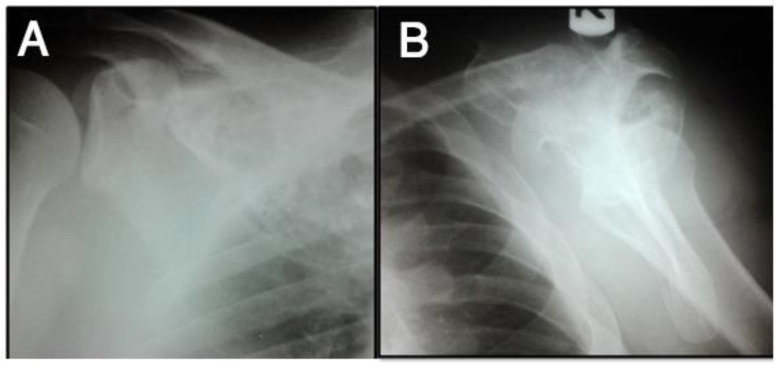
Anteroposterior (A) and oblique (B) views of right scapula of the same patient at recurrence (11 years later) show well-defined expansile lytic lesion with slightly sclerotic borders

**Figure 3:**
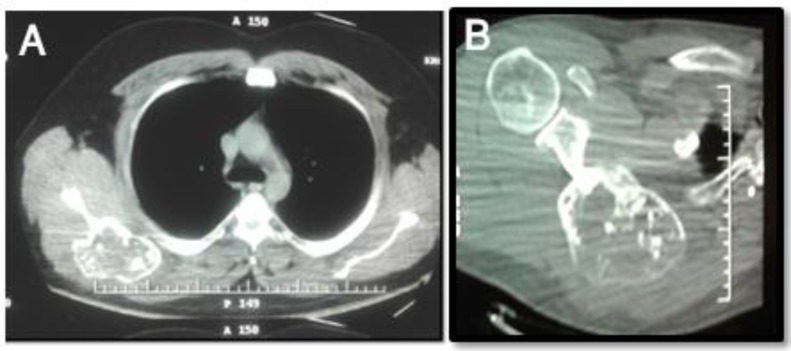
Computed Tomography images of right scapular recurred mass

**Figure 4:**
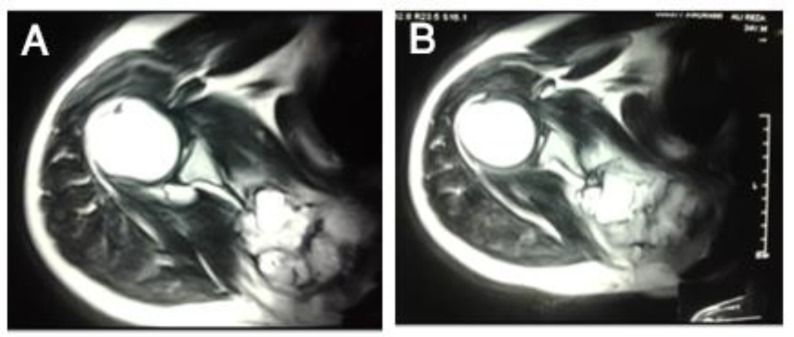
Magnetic Resonance Imaging of right shoulder at recurrence showing a large expansile hypersignal mass in medial aspect of supraspinatus muscle, within the body of scapula below the level of clavicle

He refused to take any treatment since then when he referred back with a huge scapular firm mass measuring 10×10 cm, palpated at the right upper back on physical examination with painful movements of the right shoulder. Radiographs (Figure 2), Computed Tomography (Figure 3), and Magnetic Resonance Imaging (Figure 4) showed a huge bone mass of right scapula with cortical destruction and extensive soft tissue involvement.

**Figure 5:**
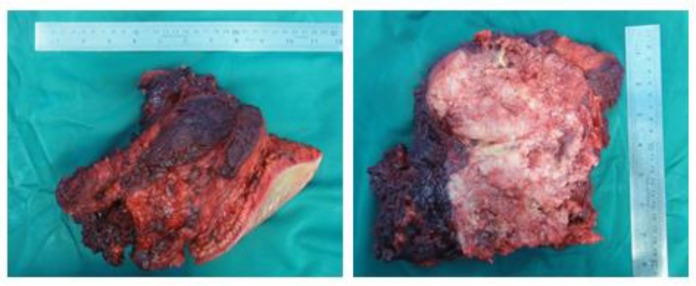
Scapulectomy specimen at recurrence, a large mass with irregular infiltrating borders and fish-fleshy appearance on sections

The patient underwent partial scapulectomy. Received surgical specimen, M: 21×16×11 cm, consisting of skeletal muscle bulk, partially covered by skin M: 14×4 cm, and a portion of scapula M: 8×8×1 cm. The skin exhibited a scar at the site of the previous biopsy. Gross inspection of the specimen sections revealed a large creamy-brown soft and fleshy mass in the scapula extending into peripheral soft tissue M: 11×10 ×7 cm. The tumor was resected with a one-centimeter safe margin (Figure 5). 

**Figure 6:**
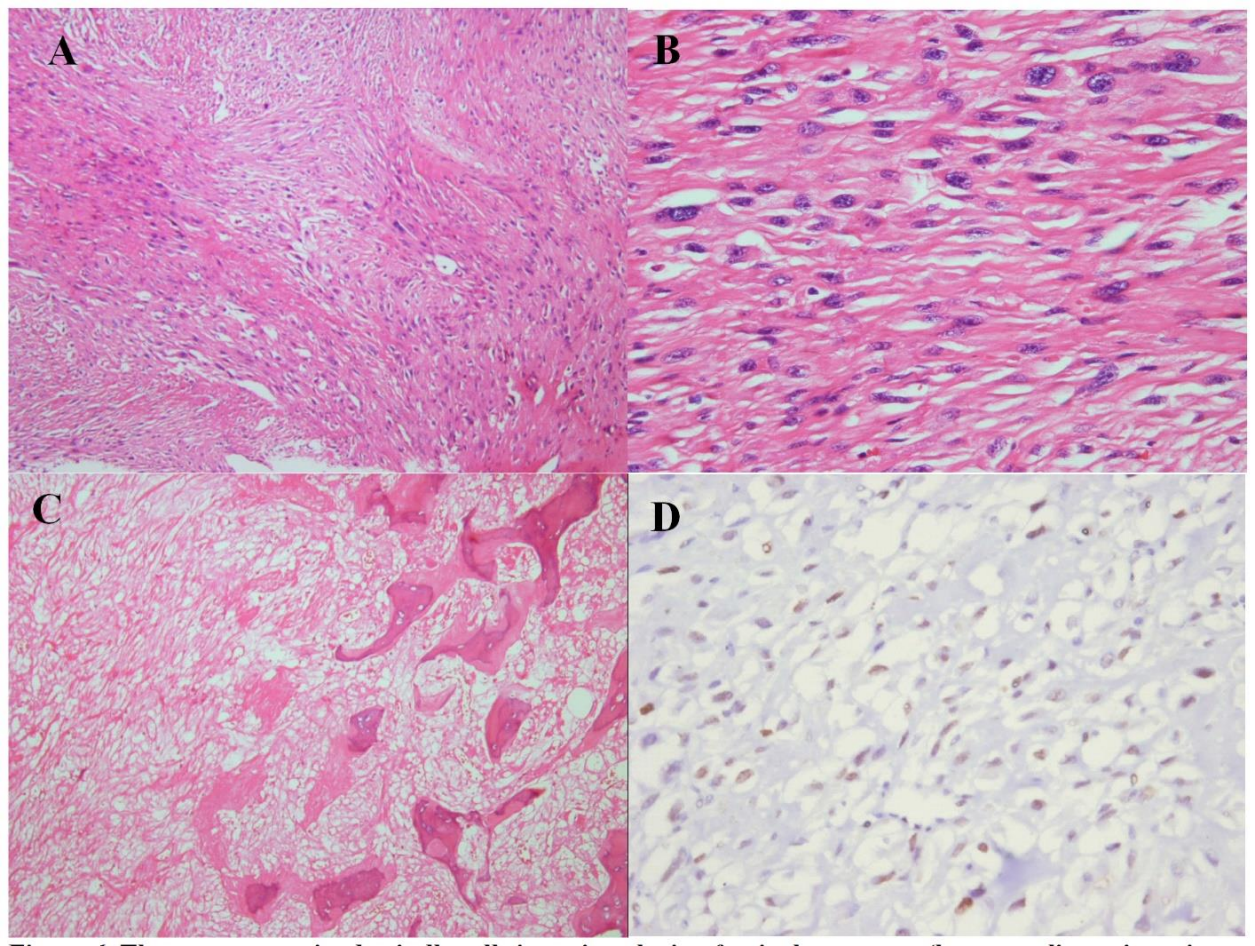
Microscopic slides of recurred mass. The tumor contains spindle cells in an interlacing fascicular pattern (H & E stain, ×200) (A). High-power view of spindle cells, showing pleomorphism and hyperchromasia (H & E stain, ×400) (B). Osteoid tissue, indicating the transformation to osteosarcoma, with foci of necrosis (H & E stain, ×200) (C). P53 antibody indicated a positive staining in almost 40% of spindle tumor cells (immunohistochemistry, ×400) (D)

Microscopic evaluation of the tumor showed a cellular neoplastic tissue composed of highly pleomorphic spindle-shaped cells arranged in fascicles with foci of necrosis and osteoid formation. Tumor cells have eosinophilic cytoplasms, vesicular nuclei, prominent nucleoli, and show marked nuclear atypia and frequent mitotic figures. The histopathologic diagnosis was high-grade osteosarcoma (Figure 6).

**Case 2**. 

**Figure 7:**
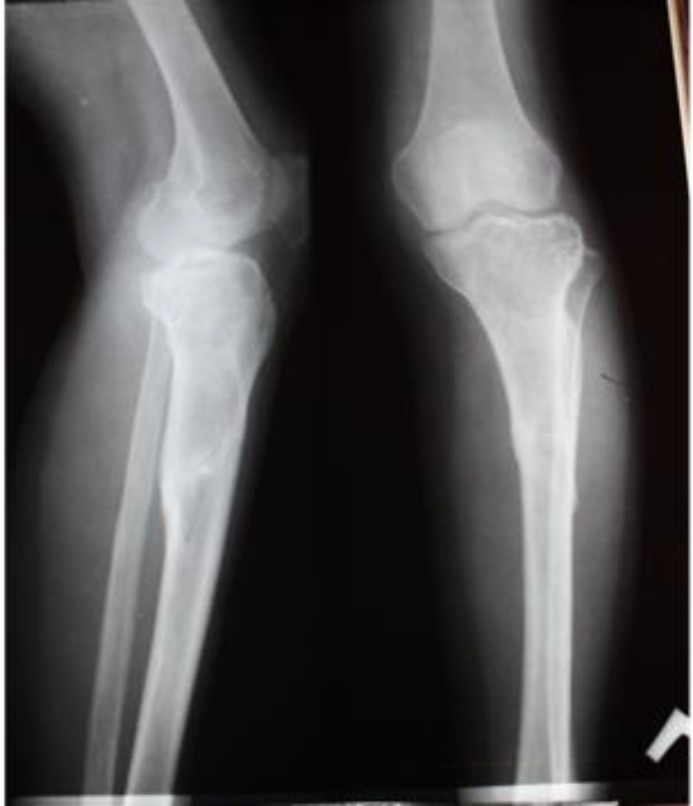
Plain radiograph of a 32-year old female with left knee GCTOB at the first presentation shows lytic expansile well-defined meta-diaphyseal lesion extending to the subarticular region

**Figure 8:**
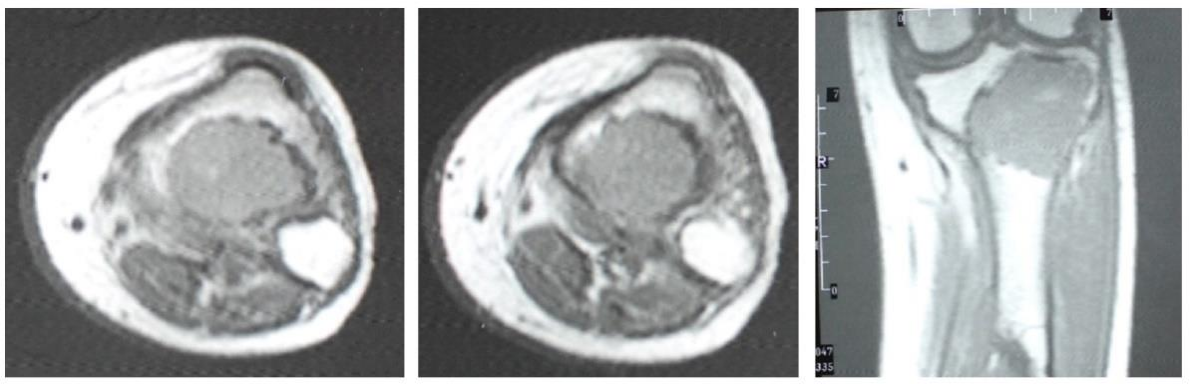
T1W Magnetic Resonance images show low signal expansile bony lesion with subarticular extension

**Figure 9:**
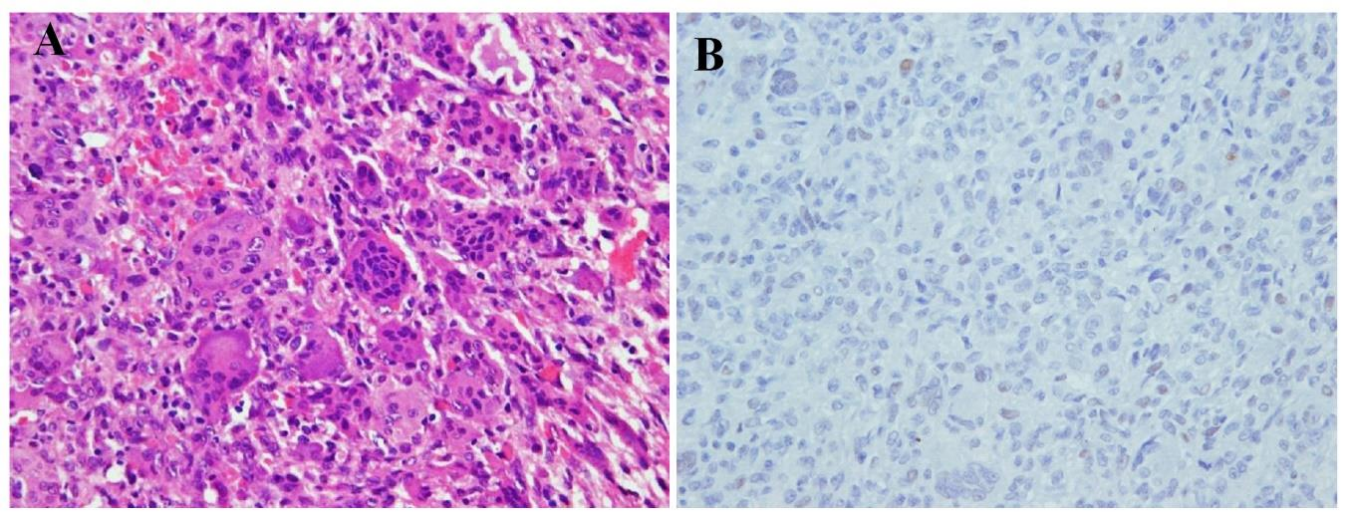
Microscopic slides of the primary lesion. The tumor consists mainly of multinucleated giant cells interspersed by stromal mononuclear cells with indistinct cytoplasmic borders. No atypia, mitotic figures, or necrosis was observed (H & E, ×20) (A). P53 staining shows less than 10% staining (immunohistochemistry, ×40) (B)

**Figure 10:**
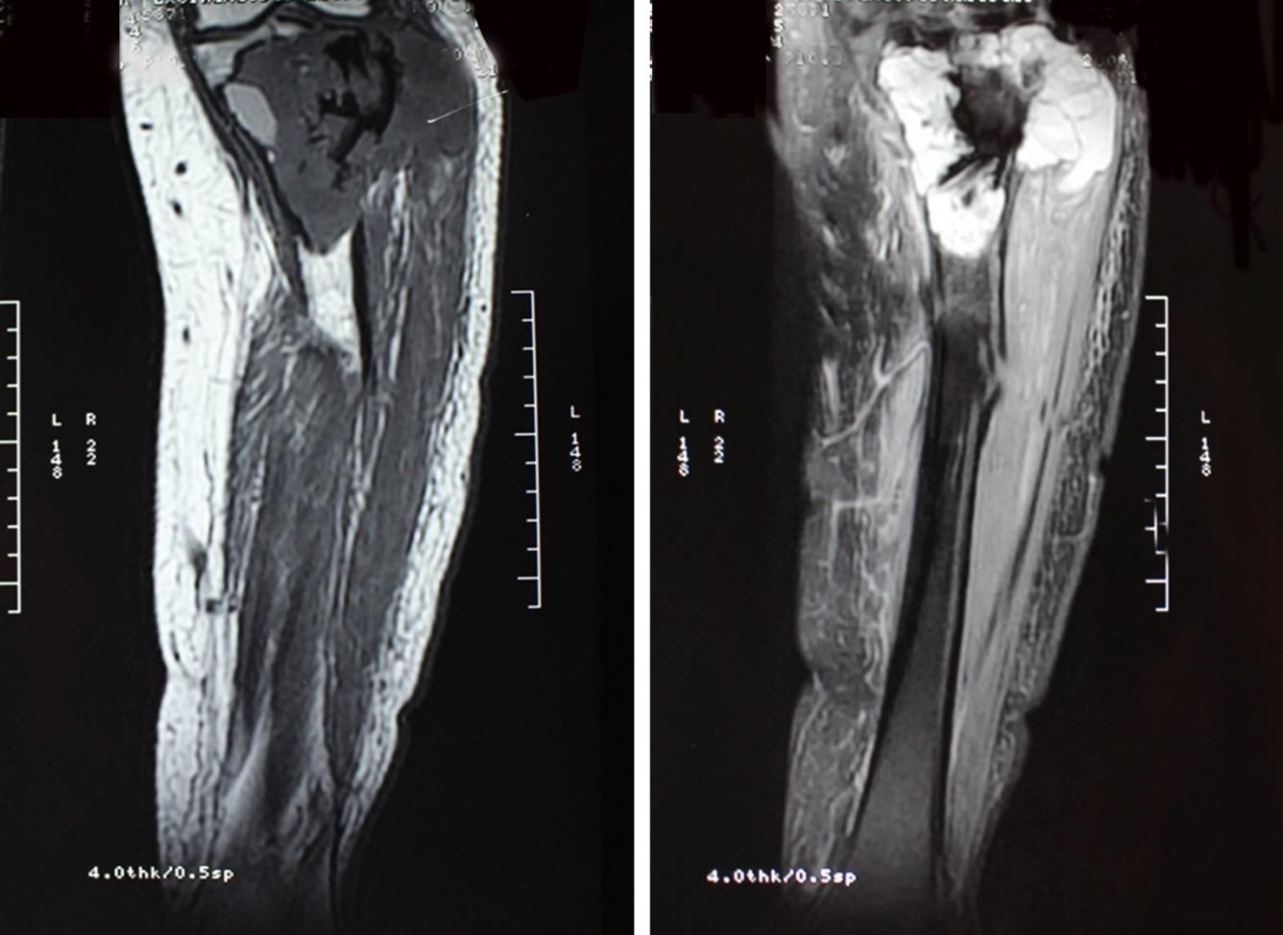
Magnetic Resonance Imaging at recurrence (6 years later), showed a destructive lesion associated with the soft tissue component and signal void center due to prior treatment with bone cement

In June 2016, a 48-year-old woman referred with a long-lasting mass at left knee since 12 years ago, which had become painful since 2 years ago but the patient had ignored it until recently, when it affected big toe range of motion. She reported a primary tumor at the same site (Figure 7, 8) 16 years before, at the age of 32 years, which was diagnosed as a conventional GCTOB (Figure 9). In August 2006, she had referred back with left knee pain and swelling. Magnetic Resonance Imaging revealed a mass at the site of the previous curettage (Figure 10) in favor of tumor recurrence. Since then, she had neglected the tumor and refused further workup or treatment.

**Figure 11:**
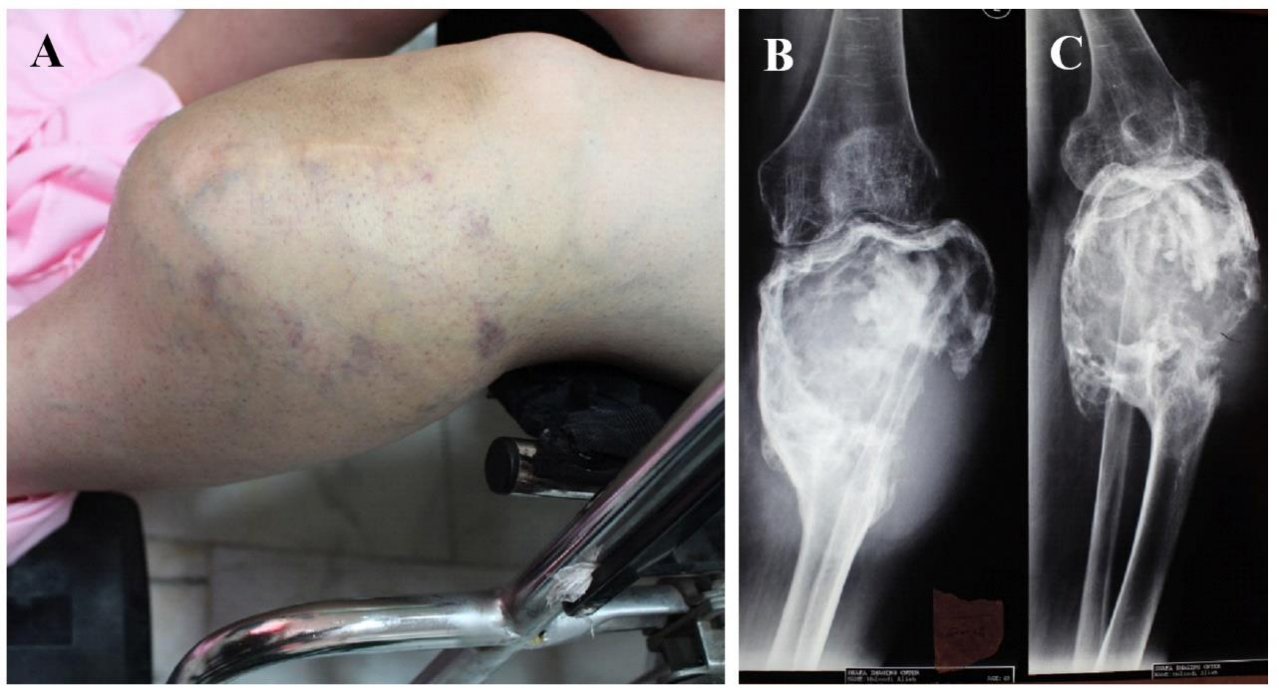
Neglected large mass below the knee 10 years after recurrence (A), plain radiographs revealed a large expansile destructive bony lesion containing mixed areas of lytic and sclerotic components associated with large soft tissue mass (B & C)

The patient suffered from restriction in first metatarsophalangeal joint extension 20 days prior to admission. Physical examination revealed a 15×10 cm palpable mass with tenderness to palpation distal to the right knee (Figure11A). She had no constitutional symptoms, nor did she have a history of trauma or infection. Plain radiographs (Figure11 B, C) demonstrated an infiltrative lytic lesion with cortical destruction in the right proximal tibia. The Chest Computed Tomography demonstrated a few nodular opacities at both lungs suggesting metastasis. 

**Figure 12:**
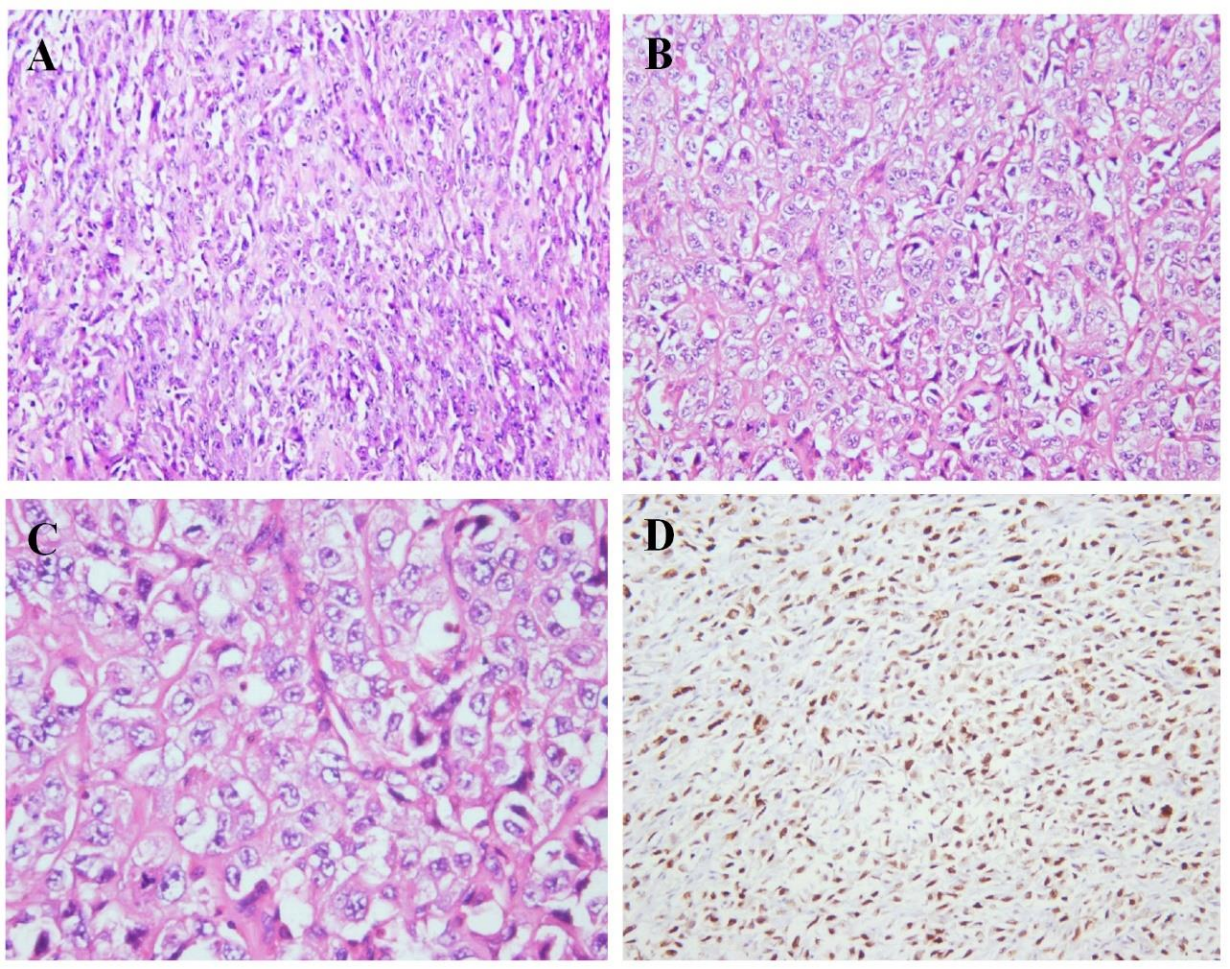
The tumor is composed of spindle tumor cells with ovaloid to elongated vesicular nuclei, arranged in sheets and vague fascicular pattern (H & E stain, × 200) (A, B). High-power view reveals pleomorphic cells with irregular nuclei borders (H & E stain, × 400) (C). P53 staining showed strong expression in about 80% of tumor cells (immunohistochemistry, × 200) (D)

Microscopic evaluation of the tumor revealed a malignant neoplasm composed of the sheets of large polymorphic cells. Tumor cells had highly atypical vesicular nuclei and frequent mitotic figures (Figure 12).

Immunohistochemical staining for p53 in both cases revealed overexpression of this marker in the malignant tumor (Fig 6B and 12D), defined as more than 10% of the tumors cells being positive, while none of the primary conventional GCTOB at first presentation showed p53 overexpression (Fig 1C and 9D).

Follow-up information revealed that one patient (case No.2) developed lung metastasis and expired, and the other is alive without tumor recurrence after receiving courses of chemotherapy.

## Discussion

Giant cell tumor of bone (GCTOB) is a benign locally aggressive neoplasm with a high local recurrence rate. Owing to the new surgical techniques, its local recurrence has now decreased to 5%-15% (10) depending on the tumor location and the type of surgical treatment (11-13). According to the literature, the majority of giant cell tumors of bone recur within the first 3 years (10,14,15) and usually not later than 5 years following primary surgery. Longer intervals between recurrences are more commonly associated with the malignant transformation (11). The sarcomatous transformation has been documented in some benign bone and tumors, including osteochondroma, chondroma, GCTOB, fibrous dysplasia, Paget’s disease, and bone infarct. The malignant transformation of GCTOB is relatively rare with a reported incidence rate ranging from1.4 to 6.6% (2,14,16). 

A primary malignant giant cell tumor is a lesion in which a typical benign GCT is seen next to the areas of synchronous high-grade sarcoma at the time of presentation. The secondary malignant transformation is defined as a metachronous high-grade sarcomatous growth superimposed on the site of a previous benign giant cell tumor, treated with either surgery or radiotherapy. 

The secondary malignant transformation of giant cell tumor is claimed to occur following radiotherapy or multiple local recurrences of conventional giant cell tumor (5), or in cases with late local recurrence (3,17). However, cases of the secondary malignant giant cell tumor treated with denosumab are on report (18). This evidence along with these two neglected cases without a history of radiation, multiple recurrences or late recurrence, suggest this hypothesis that malignant transformation may be part of the natural history of this tumor. Considering all existing hypotheses, what seems common, is that malignant changes usually take longer than 3 years to develop after the initial treatment. Therefore, in late recurrences and prolonged intervals between the emergence of the tumor and therapeutic intervention, the specimen should be carefully inspected regarding sarcomatoid changes.

The mechanism of this malignant transformation is still unknown. The losses of heterozygosity on chromosomes 9p and 17p, near the p53 locus, a tumor suppressor gene, have been reported in the cases of malignant and metastatic giant cell tumor (7-9). Some other studies suggest the involvement of GPX-1, cyclinD1, Ki-67, and CCND1 and MET genes in the malignant transformation of GCTOB (7,19). Alteration of P53 is a common mechanism of the malignant transformation and tumorigenesis in many carcinomas and bone and soft tissue sarcomas [20-22]. The p53 mutation impairs the ability of the cell to sense and repair DNA defects. Different studies have pointed to positive immunostaining of p53 in the sarcomatous transformation of GCTOB in contrast to benign tumors (4,9,23,24), as we also observed in this study. Okubo et al., studied 43 cases of GCTOB and found mutations or LOH of p53 in all three cases with the secondary malignant transformation, which also showed p53 overexpression. However, less than 20% of the conventional cases revealed non-synonymous p53 mutations, none of which showed p53 overexpression. Some others have emphasized on the prognostic value of p53 (23,24). Findings show that recurrent cases express p53 more frequently than non-recurrent ones (23) suggesting that p53 may play a role in predicting biologic behavior of GCTOB.

Histopathological findings of the malignant transformation in giant cell tumor of bone may be subtle and it may be difficult to diagnose in some cases. It is not possible to predict local aggressiveness and metastatic potential of GCTOB from the histomorphologic appearance alone. Thus finding a predictive biomarker for the malignant transformation would be a great diagnostic tool in this regard. Some studies suggest Ki-67 proliferative index and p53 positivity as prognostic markers to predict the clinical behavior of GCTOB. Our findings show overexpression of p53 in the secondary sarcomatous lesion, indicating that p53 might play a role in the malignant transformation of GCTOB.
